# Transient Anti-Phospholipid Antibodies in Two Patients With COVID-19

**DOI:** 10.7759/cureus.13026

**Published:** 2021-01-31

**Authors:** Nino Balanchivadze, Peter Xie, Philip Kuriakose, Bernd Barthel, Vrushali Dabak

**Affiliations:** 1 Hematology and Medical Oncology, Henry Ford Health System, Detroit, USA

**Keywords:** covid-19, venous thromboembolism, anti-phospholipid antibodies, b-cell proliferation

## Abstract

We report two cases of coronavirus disease 2019 (COVID-19) in patients who developed pulmonary embolism and transient anti-phospholipid antibodies. At the time of presentation with acute pulmonary embolism, both patients had leukocytosis and increased levels of anti-cardiolipin antibodies, which resolved at testing 12 weeks after initial presentation. Studying cases of pulmonary embolism and increased anti-phospholipid antibodies in the context of COVID-19 could be one of the factors for elucidating the possible connection between severe acute respiratory syndrome coronavirus 2 infection, anti-phospholipid antibodies, and thrombosis.

## Introduction

Coronavirus disease 2019 (COVID-19), caused by the novel RNA beta coronavirus severe acute respiratory syndrome coronavirus 2 (SARS-CoV-2), can have many clinical and laboratory manifestations. Because SARS-CoV-2 is new to the human population, a range of immunological responses have been noted [1]. Some severely ill patients with COVID-19 have developed coagulopathies, and the relationship between a hypercoagulable state and the presence of anti-phospholipid antibodies is currently under debate [2]. We describe two patients with COVID-19 who developed pulmonary embolism and had transient anti-phospholipid antibodies, which were present during the acute illness but disappeared during recovery.

## Case presentation

A 59-year-old African American man with a history of double heterozygous state with sickle cell trait/beta thalassemia+ presented to the emergency department with shortness of breath and pleuritic chest pain. He was febrile, tachycardic, and hypoxemic on arrival and was hospitalized for further care. Diagnostic investigations showed leukocytosis (27.8 × 103/μL) with normal absolute lymphocyte count, microcytic anemia (hemoglobin 6.8 g/dL) with abnormal hemolysis markers, and a normal platelet count. Reverse-transcription polymerase chain (PCR) test from nasopharyngeal swab was positive for SARS-CoV-2. Computed tomography (CT) imaging of the chest revealed multi-lobar infiltrates and bilateral upper lobe pulmonary embolism. Anti-phospholipid antibody testing showed the presence of cardiolipin Ab IgG 33.5 GPL, cardiolipin Ab IgM 41.7 MPL, and B2 glycoprotein Ab IgG 103 SGU (Table 1). Lupus anticoagulant testing was not performed due to concern of its validity in the setting of active anticoagulation therapy.

**Table 1:** Laboratory investigation of peripheral blood showing anti-phospholipid antibodies at the time of diagnosis of pulmonary emboli and 12 weeks later.

	Cardiolipin Ab IgA, APL		Cardiolipin Ab IgG, GPL		Cardiolipin Ab IgM, MPL		B2 Glycoprotein IgA Ab, SAU	
Reference range	<12 APL		<15 GPL		<12.5 MPL		<20 SAU	
	Initial	At 12 weeks	Initial	At 12 weeks	Initial	At 12 weeks	Initial	At 12 weeks
Patient 1	<9	<9	33.5	<9	41.7	<9	103	<9
Patient 2	<9	<9	83.4	<9	136.0	<9	<9	<9

At the follow-up appointment 12 weeks after hospital discharge, the patient was found to be recovering well with resolution of leukocytosis. Repeat testing showed disappearance of anti-phospholipid antibodies. He completed a three-month course of therapeutic anticoagulation therapy for his pulmonary embolism, presumed to be provoked, and has remained asymptomatic during his follow-up appointments.

In the second case, a 41-year-old Caucasian man with no significant past medical history presented to the emergency department with five-day duration of progressive shortness of breath, productive cough, and fever. His wife had been diagnosed with COVID-19 10 days prior to his presentation. On admission to the hospital he was febrile, tachycardic, and hypoxemic (oxygen saturation 84% on room air). Diagnostic investigations revealed leukocytosis with monocytic predominance (white blood cell count, 11.5 k/UL; absolute monocyte count, 1.90 k/UL), hemoglobin and platelet counts within reference range, and he had elevated ferritin (2277 ng/mL) and lactate dehydrogenase (365 IU/L). CT of the chest revealed multi-focal infiltrates and bilateral pulmonary embolism, including a saddle embolus with evidence of right heart strain. SARS-CoV-2 PCR test via nasopharyngeal swab was negative. Anti-cardiolipin antibodies were outside of reference range, with cardiolipin Ab IgA <9.0 APL, cardiolipin Ab IgG 83.4 GPL, and cardiolipin Ab IgM 136 MPL (Table 1). Lupus anticoagulant testing was not performed. He was treated supportively for presumed COVID-19 community-acquired pneumonia. Therapeutic anticoagulation was initiated for his bilateral pulmonary embolism and he was discharged home with supplemental oxygen in stable condition.

The patient was seen in the hematology clinic three months later where he was recovering well without oxygen requirement. Repeat testing showed normalization of anti-phospholipid antibodies and resolution of leukocytosis. A test for IgG SARS-CoV-2 antibodies in peripheral blood was reactive, strongly suggesting that his initial nasopharyngeal swab had been a false negative. He completed three months of anticoagulation therapy with apixaban without any complications.

## Discussion

In the cases presented here, both patients had pulmonary embolism and showed clear presence of anti-cardiolipin and anti-beta-2 glycoprotein antibodies during acute illness that resolved in the weeks following recovery.

Anti-phospholipid antibodies have been implicated in the pathogenesis of thrombosis and pregnancy morbidity in patients with anti-phospholipid syndrome [3]. Also, case reports of transient anti-phospholipid-induced thromboses in patients with viral infections, such as acute cytomegalovirus infections have been observed [4], with different proposed explanations.

Severe COVID-19 infection has been shown to be associated with increased risk of thromboembolism [5]. Both venous and arterial thromboses have been described [6]. Autopsy findings of microthrombi in various organs is suggestive of its pathogenesis in multi-organ failure in patients with COVID-19 [7]. The exact mechanism of COVID-19-related thrombosis is not clear but mechanisms that lead to thrombosis with infection and inflammation are likely contributory [8]. It is believed that severe inflammatory response and subsequent release of inflammatory cytokines leads to endothelial activation and dysfunction, expression of tissue factor and other procoagulants, with the end result of thrombin generation and fibrin clot formation [9]. Whether transient anti-phospholipid antibodies play a role in development of thrombosis in patients with COVID-19 is unclear.

We suspect that the transient anti-phospholipid antibodies seen in our patients were reactive in nature and perhaps had developed from antigenic stimulation caused by SARS-CoV-2 virus and a massive inflammatory response. We wonder whether transient anti-phospholipid antibody production contributed to the development of pulmonary emboli. The patient described in the first case had abnormal hemoglobin electrophoresis that revealed a double heterozygous state with sickle cell trait/beta thalassemia+, possibly placing him at higher risk of venous thromboembolism and explaining his severe anemia at presentation. For the patient described in case two, we could not identify any other triggering events for his hemodynamically significant pulmonary embolism.

We hypothesize that COVID-19 had contributed to development of venous thromboembolism in both patients, with transient anti-phospholipid antibodies possibly playing a role in pathogenesis. Studying the relationship between anti-phospholipid antibodies and thromboembolic complications in patients with COVID-19 will be important for understanding the multiple pathogenic mechanisms of SARS-CoV-2 infection and to inform treating physicians about the many clinical complications that this problematic virus may cause.

## Conclusions

Anti-phospholipid antibody testing might be beneficial in individuals with COVID-19 and venous thromboembolism for better understanding of thrombotic complications. The cause and effect relationship of transient immunological responses with the development of pulmonary embolism in patients with COVID-19 and the long-term implications of these transient findings are uncertain and require investigation.
